# Supplementary material for: Content-rich biological network constructed by mining PubMed abstracts
Source: BMC Bioinformatics. 2004 Oct 8;5:147. doi: 10.1186/1471-2105-5-147 (PMC528731; doi:10.1186/1471-2105-5-147)
Supplement: Additional File 2 — The original results of the above study (non-essential files are deleted to keep the file size under the limit set by BMC bioinformatics). [file 1471-2105-5-147-S2.bz2 › chilibotAdditionalFile2/dip10/502ID7878020E10668/html/TGFB3_TGFBR2.html]

 


 **TGFB3** and **TGFBR2** 
  
Found 31 abstracts in PubMed, retrieved 10.  
 

 What does Google say? 
 PDF only 
| .edu only 

---

**Interactive relationship** (e.g. stimulation, inhibition, etc)

**Non-interactive relationship** (e.g. studied together, co-existance, homology, etc.)

- Here, we present the 2.15 A crystal structure of the extracellular ligand binding domain of the human TGF beta type II receptor  [ **TGFBR2** ]  ecTbetaR2 in complex with human TGF beta3  [ **TGFB3** ] .  Ref: 11850637 Nat Struct Biol, 2002
- Immunohistochemical localization of TGF beta type II receptor  [ **TGFBR2** ]  and TGF beta3  [ **TGFB3** ]  during palatogenesis in vivo and in vitro.  Ref: 9727838 Int J Dev Biol, 1998
- Messenger RNA was isolated from the nucleus pulposus using oligo 25 superparamagnetic beads and probing with gene specific primers in RT PCR. mRNA coding for TGF alpha, EGF, TGF beta1 and TGF beta3  [ **TGFB3** ]  or the EGF receptor and TGF beta type II receptor  [ **TGFBR2** ]  was found only occasionally.  Ref: 10615986 J Bone Joint Surg Br, 1999
- We therefore studied the expression of UPA, UPAR, IGF2R, ALK5,  **TGFBR2** , TGFBR3, ENG, ALK1, TGFB1, TGFB2, and  **TGFB3**  in a series of 14 pancreatic carcinoma cell lines.  Ref: 11408925 Int J Oncol, 2001
